# Sarcopenia in Older Adults

**DOI:** 10.3390/jcm8111844

**Published:** 2019-11-02

**Authors:** David Scott

**Affiliations:** 1 Department of Medicine, School of Clinical Sciences at Monash Health, Monash University, Clayton 3168, Victoria, Australia; david.scott@monash.edu; 2 Department of Medicine and Australian Institute of Musculoskeletal Science, Melbourne Medical School—Western Campus, The University of Melbourne, St. Albans 3021, Victoria, Australia

Sarcopenia was first described in 1988 as the age-related decline of skeletal muscle mass [1]. In the last decade, several international groups have developed operational definitions of sarcopenia which incorporate assessments of muscle function in addition to muscle mass in sarcopenia case-finding guidelines [2,3,4,5]. Nonetheless, recent changes to these recommendations demonstrate that there is significant work to be done in order to achieve an international consensus on the methods to diagnose and treat sarcopenia in clinical settings [6,7]. Indeed, while sarcopenia is now recognised with its own International Classification of Diseases, 10th Revision, Clinical Modification (ICD-10-CM) code (M62.84) [8], few health care professionals know how to diagnose it, include it as part of their clinical practice, or even recognise it as a disease, [9,10]. This Special Issue of the *Journal of Clinical Medicine* was established with a view to increase clinician awareness of the important clinical consequences of sarcopenia and improve understanding of diagnosis and treatment strategies. 

Computed tomography (CT) provides precise estimates of muscle quality and so may be useful for case-finding in sarcopenia, but barriers to this method include high costs, lack of availability and ionising radiation doses. Ultrasound represents a potentially cost-effective, accessible and safe method of measuring muscle quality and Harris-Love and colleagues demonstrate in this special issue that rectus femoris echogenicity assessed by ultrasound is significantly correlated with intra- and inter-muscular adipose tissue estimates from CT, and has similar associations with muscle function and cardiometabolic outcomes [11]. For clinicians without requisite expertise or access to equipment to perform muscle mass assessments, it is possible that questionnaires can be useful in identifying patients at risk of sarcopenia. The SarQoL^®^ (Sarcopenia and Quality of Life), is a validated quality of life (QoL) questionnaire which can discriminate sarcopenic and non-sarcopenic patients and has already been translated into several languages. In this issue, a study on the validation of a Polish version of the SarQoL^®^ is described, highlighting the potential utility of this instrument for identifying individuals at risk of sarcopenia, as well as its impact on QoL, across a number of countries worldwide [12]. Physical function assessments are also simple to perform in clinical settings and may be most appropriate to identify sarcopenia cases in obese older adults who, owing to their larger muscle mass, may generally exceed cut-off points for low muscle mass. Mesinovic and colleagues demonstrate associations between the metabolic syndrome and poorer muscle function despite the generally larger muscle size in overweight and obese older individuals, indicating accelerated loss of muscle quality is common in this population [13].

Sarcopenia is generally considered a disease of ageing but there is no doubt that risk of developing sarcopenia is increased by numerous medical conditions. Moreover, the presence of sarcopenia appears to consistently impact on prognosis of those with existing comorbidities. Indeed, the review by Han and colleagues demonstrates that sarcopenia is associated with increased progression of chronic disease, postoperative complications, length of hospital stay, all-cause mortality and cognitive impairment [14]. The effects of sarcopenia on cognitive impairment are supported by an original study of community-dwelling older adults residing in rural areas of Korea which reported that physical frailty was significantly associated with poor cognitive function [15]. Liver disease is also associated with weight loss and physical frailty, and a population-based study reported that non-alcoholic fatty liver disease was more common in Korean adults with low muscle mass determined by CT, regardless of their obesity status [16]. Similarly, a study of Japanese patients with chronic liver disease reported that 50% had low muscle mass, either alone or combined with low muscle strength. Interestingly, low serum zinc concentration was an independent predictor of sarcopenia in these patients suggesting zinc supplementation warrants further investigation as a strategy to prevent muscle declines in chronic liver disease [17]. 

Indeed, many researchers internationally are focused on developing translatable lifestyle interventions to improve physical function in older adults with sarcopenia and other conditions associated with functional decline. The potential to translate such programs to the community may be improved by providing interventions which can be performed in the home. Mair and colleagues describe a six-week low-volume, home-based weighted step exercise program in eleven women aged 65–74 years, which resulted in improved lower-limb muscle power and physical function [18]. However, in Greek older adults with sarcopenia, patients randomised to supervised group-based exercise for 24 weeks had greater improvements in muscle mass, strength and physical performance compared with patients performing home-based exercise [19], indicating that supervised exercise is likely most beneficial for preventing and treating sarcopenia. Blood-flow restriction resistance training needs to be performed in supervised settings due to potential safety issues but has shown promise in improving muscle mass and function. It has also been suggested to potentially reduce training-associated joint pain, and Harper and colleagues explored this in older adults with physical limitations and symptomatic knee osteoarthritis randomised to 12 weeks of lower-body low-load resistance training with blood-flow restriction or moderate-intensity resistance training. Somewhat surprisingly, functional changes appeared to favour moderate-intensity resistance training, although fewer reports of knee pain were observed for the blood-flow restriction group suggesting it may be a safe method to deliver exercise in those with joint pain [20]. Another pilot study in this Special Issue explored the use of three different antihypertensive medications in combination with aerobic and resistance exercise for improving physical function in older adults with hypertension [21]. The authors reported acceptable feasibility and safety for this intervention which can inform the design of larger randomised controlled trials to determine the effects of combined pharmacotherapy and exercise in older adults.

In addition to medications, the use of nutritional supplements to enhance exercise outcomes is an area of great interest to researchers, clinicians and the general public. Buckinx and colleagues report the effects of a 12-week intervention combining supplementation of the non-proteinogenic amino acid l-citrulline with high-intensity interval training in older adults with obesity and low muscle strength. The results demonstrated that supplementation with l-citrulline, compared with placebo, resulted in greater exercise-induced improvements in upper-limb muscle strength and walking speed in this population [22]. While numerous nutritional supplements have been explored for treatment of sarcopenia, some of the most compelling evidence exists for creatine. To close this Special Issue, Candow and colleagues present an elegant review on creatine’s association with muscle, bone and inflammation during ageing. They highlight the growing evidence that creatine supplementation is safe, and with or without exercise, can increase muscle mass and strength, and potentially reduce risk of falls in older adults [23]. 

In summary, this Special Issue provides important new evidence on methods to diagnose and treat sarcopenia, and its relationships with common chronic diseases in older adults. It is our hope that this novel information will serve as an important resource for researchers, and aid health professionals to increase the current low level of attention to sarcopenia in clinical settings. 
